# Appropriate definition of non-metastatic castration-resistant prostate cancer (nmCRPC) and optimal timing of androgen receptor signaling inhibitor (ARSI)

**DOI:** 10.1007/s10147-024-02549-5

**Published:** 2024-06-10

**Authors:** Kazuhiro Matsumoto, Takeo Kosaka, Toshikazu Takeda, Keishiro Fukumoto, Yota Yasumizu, Nobuyuki Tanaka, Shinya Morita, Ryuichi Mizuno, Hiroshi Asanuma, Mototsugu Oya

**Affiliations:** Department of Urology, Keio University School of Medicine, 35 Shinanomachi, Shinjuku-Ku, Tokyo, 160-8582 Japan

**Keywords:** Prostate cancer, nmCRPC, Androgen receptor signaling inhibitor, ARSI, m0CRPC

## Abstract

**Background:**

Defined by rising PSA levels under androgen deprivation therapy (ADT) despite no visible metastases on conventional imaging, non-metastatic castration-resistant prostate cancer (nmCRPC) represents a complex clinical challenge. A significant subset of these patients rapidly develops metastatic disease, negatively impacting survival. We examined the difference in prognosis of nmCRPC patients according to the timing of therapeutic interventions with androgen receptor signaling inhibitor (ARSI).

**Methods:**

We examined 102 nmCRPC patients treated with ARSI. We divided patients according to their PSA levels when ARSI was administered: Cohort A (PSA 0.5–2.0 ng/mL), Cohort B (PSA 2.0–4.0 ng/mL), and Cohort C (PSA > 4.0 ng/mL). Utilizing the Kaplan–Meier method for survival analysis, our analytical starting point was the moment when PSA levels exceeded 0.5 ng/mL post-ADT nadir, ensuring a fair comparison and minimizing lead-time bias.

**Results:**

After excluding 5 patients whose PSA nadir after ADT > 0.5 ng/mL, patient distribution across Cohort A, Cohort B, and Cohort C was 32, 24, and 41 patients, respectively. Kaplan–Meier survival analysis highlighted a 2-year metastasis-free survival rate of 97% for Cohort A, 87% for Cohort B, and 73% for Cohort C. A marked statistical difference emerged when comparing Cohort A with Cohorts B and C, with a p-value of 0.043.

**Conclusion:**

The timely initiation of ARSI is paramount in nmCRPC management. Our findings strongly advocate for consideration of ARSI administration in nmCRPC patients before their PSA levels exceed 2.0 ng/mL. Our results indicated a PSA threshold of 1.0 ng/mL for nmCRPC definition which is more reasonable to administer ARSI without delay.

## Introduction

Prostate cancer, primarily driven by androgens, is a significant male malignancy. Although androgen deprivation therapy (ADT) remains an effective initial treatment approach, the majority of patients develop castration resistance over time. Non-metastatic castration-resistant prostate cancer (nmCRPC) represents a clinical stage in which patients show a rise in prostate-specific antigen (PSA) levels despite maintaining castrate levels of testosterone, but do not yet show radiographic evidence of metastasis. Roughly one-third of patients with prostate cancer receiving ADT due to biochemical recurrence post-local treatment will develop CRPC. Alarmingly, the average duration between the diagnosis of castration resistance and mortality is only 2.5 years [1].

Most nmCRPC patients are asymptomatic or show minimal symptoms at diagnosis, a result of the preceding local treatments. Particularly, those with short PSA doubling times and elevated baseline PSA levels possess a heightened risk for distant metastases and eventual prostate cancer-related death [2]. The major challenge in managing these patients is balancing therapeutic efficacy while preserving quality of life.

Several clinical trials, including PROSPER [3], SPARTAN [4], and ARAMIS [5], have investigated therapeutic interventions for this patient population using androgen receptor signal inhibitors (ARSI). The definitions of nmCRPC in those trials were consistent, focusing on the elevation in PSA values and the rapidity of the PSA doubling time, despite ongoing ADT. All trials used a threshold PSA value of ≥ 2.0 ng/mL and a PSADT of ≤ 10 months to define their study populations. These definitions highlight the critical role of PSA kinetics in assessing disease progression and potential therapeutic interventions in the nmCRPC setting.

The Prostate Cancer Working Group (PCWG) plays a pivotal role in shaping guidelines and criteria for the design and interpretation of clinical trials for prostate cancer. One of the critical indicators of prostate cancer progression, especially in patients with nmCRPC, is the PSA level. Historically, a PSA level of 2.0 ng/mL or above has been considered an indicator of disease progression, as applied in the clinical trials above [6]. However, recent shifts in therapeutic strategies, coupled with the emergence of newer treatment agents, have necessitated a more nuanced approach. To this end, the PCWG3 has redefined the PSA threshold from 2.0 to 1.0 ng/mL, thereby potentially allowing for earlier therapeutic interventions and a more detailed evaluation of the disease state [7].

In this study, we examined the difference in prognosis of nmCRPC patients according to the timing of therapeutic interventions with ARSI. Our results confirm the validity of a PSA threshold of 1.0 ng/mL for the nmCRPC definition proposed by the PCWG3.

## Materials and methods

After receiving institutional review board approval, we retrospectively analyzed 102 consecutive patients who had received ARSI including enzalutamide, apalutamide, darolutamide, and abiraterone for nmCRPC during the period from 2014 to 2022 at Keio University Hospital. When administrating ARSI, castrate levels of testosterone (less than 50 ng/mL) were confirmed in all patients. Patients with de novo neuroendocrine prostate cancer were excluded.

Patients were stratified into three cohorts based on their PSA levels at the commencement of ARSI: Cohort A (PSA 0.5–2.0 ng/mL), Cohort B (PSA 2.0–4.0 ng/mL), and Cohort C (PSA > 4.0 ng/mL). We utilized the Kaplan–Meier method to assess survival outcomes. When examining the prognosis of the whole cohort, we set the starting point at the onset of ARSI administration for nmCRPC. Whereas, when comparing the difference of prognosis according to the timing of ARSI administration, we designated the starting point not at the onset of ARSI administration, but when PSA levels surpassed 0.5 ng/mL after the ADT nadir to prevent the lead-time bias. Consequently, five patients whose PSA nadir after ADT was higher than 0.5 ng/mL were excluded from the analysis. All patients underwent imaging studies when they were diagnosed with nmCRPC. After that, almost every year we performed imaging studies. We also considered additional imaging studies at the timing of every doubling of the PSA level. The endpoint was the appearance of distant metastasis detected by any imaging studies including computed tomography (CT), bone scintigraphy, and magnetic resonance imaging (MRI). Differences among the groups were analyzed using the log-rank test. The median follow-up period after ARSI administration was 2.6 years (range, 0.2–8.2), and after PSA > 0.5 ng/mL was 3.2 years (range, 0.4–11.9).

PSA doubling time provides an estimation of the time it would take for the PSA to double at the observed rate of growth [8]. In this study, it was calculated using the two timings of PSA (the first PSA after PSA > 0.5 ng/mL and that at ARSI administration) and their interval (in months).

Differences in continuous variables between two groups were analyzed using the Mann–Whitney U test. Chi-squared test was used to analyze the difference in numbers of patients between two groups. In all analyses, *p* < 0.05 was considered significant. All analyses were performed using R software (version 3.0.2).

## Results

**Fig. 1:**
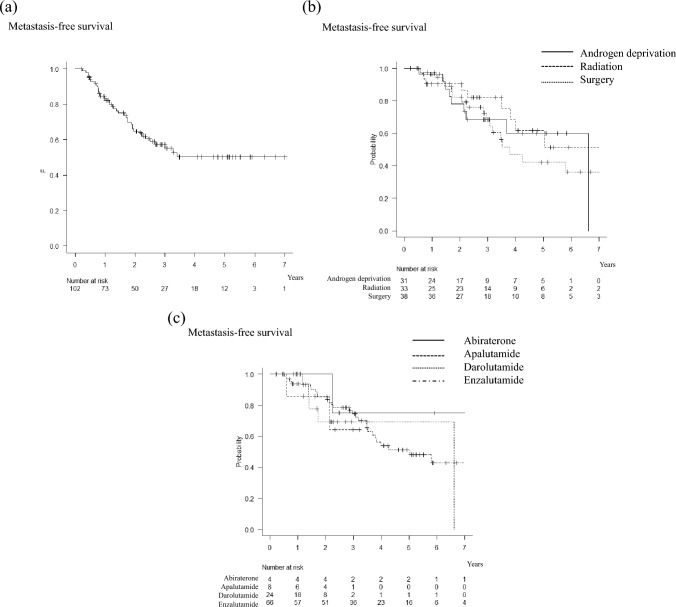
**a** During the follow-up, distant metastasis manifested in 39 patients, and 8 of them died due to prostate cancer. In all 102 patients, the 2- and 5-year metastasis-free survivals after ARSI administration were 65%, and 50%, respectively. **b** Regarding the primary treatment for prostate cancer, surgery, radiation, and ADT were performed for 38, 33, and 31 patients, respectively. There was no statistically significant difference in metastasis-free survival curves among the primary treatments. **c** To target nmCRPC, enzalutamide, darolutamide, apalutamide, and abiraterone were administered for 66, 24, 8, and 4 patients, respectively. There was no statistically significant difference in metastasis-free survival curves among the four kinds of ARSI

During the follow-up, distant metastasis manifested in 39 patients, and 8 of them died due to prostate cancer. The metastasis-free survival curve for all 102 patients after ARSI administration is shown in Fig. 1a. A diverse primary treatment landscape was evident with surgery, radiation, and ADT, constituting primary therapies for 38, 33, and 31 patients, respectively. There was no statistically significant difference in the metastasis-free survival curves among the primary treatments, as shown in Fig. 1b. For targeting of nmCRPC, the ARSI choices were enzalutamide (66 patients), darolutamide (24), apalutamide (8), and abiraterone (4). The metastasis-free survival curves of these four types of ARSI are shown in Fig. 1c.

**Fig. 2:**
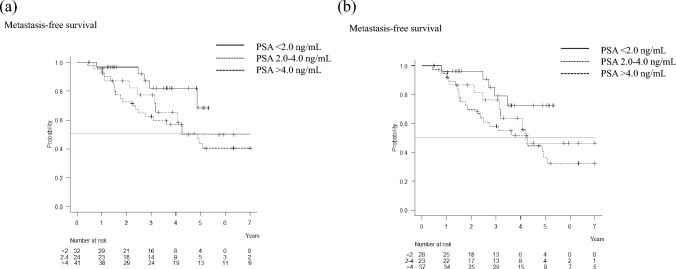
**a** Regarding the timing of ARSI administration, Kaplan–Meier survival analysis demonstrated that a 2- and 5-year metastasis-free survival rate of 97% and 68% for Cohort A, 87% and 50% for Cohort B, and 73% and 44% for Cohort C, respectively. The starting point was set when PSA levels surpassed 0.5 ng/mL after the ADT nadir. There was a significant statistical difference when comparing Cohort A with Cohorts B and C, with a p-value of 0.043. **b** Excluding 9 patients whose PSA doubling time was longer than 10 months, 2- and 5-year metastasis-free survival rates were 96% and 73% for Cohort A, 87% and 46% for Cohort B, and 70% and 36% for Cohort C, respectively. The starting point was set when PSA levels surpassed 0.5 ng/mL after the ADT nadir. A significant statistical difference was observed when comparing Cohort A with Cohorts B and C, with a *p*-value of 0.048

After excluding five patients whose PSA nadir after ADT > 0.5 ng/mL, patient distribution across Cohort A (PSA 0.5–2.0 ng/mL), Cohort B (PSA 2.0–4.0 ng/mL), and Cohort C (PSA > 4.0 ng/mL) was 32, 24, and 41, respectively. Table 1 summarizes the difference in patients’ backgrounds among the 3 cohorts, with no statistical difference observed. Kaplan–Meier survival analysis highlighted a 2-year metastasis-free survival rate of 97% for Cohort A, 87% for Cohort B, and 73% for Cohort C (Fig. 2a). The median metastasis-free times for Cohort B and Cohort C were 4.2 and 4.3 years, respectively. However, that in Cohort A was not reached. Notably, a marked statistical difference emerged when comparing Cohort A with Cohorts B and C, with a *p*-value of 0.043.

**Table 1:** Difference in patients’ backgrounds among the 3 cohorts

(Timing of ARSI administration) Median PSA (range)	Cohort A (PSA 0.5–2.0 ng/mL) 1.11 ng/mL (0.51–1.97)	Cohort B (PSA 2.0–4.0 ng/mL) 3.05 ng/mL (2.01–4.00)	Cohort C (PSA > 4.0 ng/mL) 7.40 ng/mL (4.05–28.5)	*p*-value		
				A vs. B	B vs. C	A vs.C
N	32	24	41			
Median Age (range)	77.4 years (64–89)	75.3 years (58–89)	81.3 years (55–94)	0.529	0.081	0.099
Median PSA at diagnosis (range)	10.7 ng/mL (2.0–192)	20.0 ng/mL (4.9–186)	14.4 ng/mL (5.0–194)	0.353	0.653	0.240
Clinical stage at diagnosis				0.890	0.451	0.193
cT1-3aN0M0	13 (43%)	11 (50%)	22 (61%)			
cT3bN0M0	9 (30%)	6 (27%)	5 (14%)			
cT2-3N1M0	8 (27%)	5 (23%)	7 (19%)			
Unknown(M0)	2	2	5			
Gleason grade group at diagnosis				0.600	0.302	0.655
1–3	13 (43%)	10 (42%)	18 (53%)			
4	5 (17%)	2 (8%)	5 (15%)			
5	12 (40%)	12 (50%)	10 (29%)			
Unknown	2		7			
Primary treatment				0.736	0.977	0.794
Surgery	12 (38%)	9 (38%)	15 (37%)			
Radiation	12 (38%)	7 (29%)	13 (32%)			
Androgen deprivation	8 (25%)	8 (33%)	13 (32%)			
Median PSA doubling time (range)	6.6 months (1.0–20.5)	4.5 months (1.3–13.4)	6.2 months (1.0–20.7)	0.275	0.308	0.859

*ARSI* androgen receptor signaling inhibitor; *PSA* prostate-specific antigen

Focusing on the patients with faster PSA doubling time, we excluded 9 patients (4 patients in Cohort A, 1 in Cohort B, and 4 in Cohort C) whose PSA doubling time was longer than 10 months, and we again compared the cohorts. Similar results were obtained (2-year metastasis-free survival rate of 96% for Cohort A, 87% for Cohort B, and 70% for Cohort C) (Fig. 2b). Median metastasis-free intervals for Cohort B and Cohrt C were 4.2 and 4.3 years, respectively. However, that in Cohort A was not reached. Again, a statistical difference was observed when comparing Cohort A with Cohorts B and C, with a *p*-value of 0.048.

## Discussion

Radical prostatectomy (RP), when utilized as an initial remedial approach, can offer substantial control over cancer in a majority of males grappling with clinically confined maladies. However, it is estimated that around 35% of males will undergo a discernible elevation in PSA levels [9]. The consistent application of PSA tracking allows early detection of postoperative recurrences without any radiological indicators of cancer. The recurrence of biochemical elements (BCR) is typically characterized by a symptom-free rise in postoperative PSA levels to a point exceeding 0.2 ng/mL, [10] predominantly resulting from localized reoccurrences. Hence, the principal therapeutic strategy for addressing BCR is applying salvage radiation therapy (RT) (a secondary line of treatment) to the prostatic bed. The prevailing body of research indicates that initiating salvage RT while PSA values are ≤ 0.5 ng/mL corresponds with enhanced biochemical progression-free survival [11, 12]. Conversely, for those experiencing a resurgence in PSA post salvage RT, enduring ADT emerges as a tertiary line of treatment. Our prior studies indicated that commencing salvage ADT (tertiary line of treatment) prior to PSA levels surpassing 1.0 ng/mL served as a pivotal component in averting the development of CRPC, particularly in high-risk subjects with accelerated PSA doubling time [13].

Regrettably, a subset of such cases experiences a resurgence in PSA levels, even when imaging studies reveal no distant metastasis, a condition referred to as nmCRPC. The nmCRPC state can also arise when salvage ADT is initiated subsequent to initial treatment with radiation therapy or initial ADT for localized prostate cancer. Within the scope of nmCRPC, several pivotal factors, including baseline PSA level, PSA velocity, and PSA doubling time, are intimately associated with vital patient outcomes including the time to initial bone metastasis, bone metastasis-free survival, and overall survival [2, 14].

The PCWG has been instrumental in establishing definitions and guidelines related to prostate cancer. Initially, the PCWG proposed that a PSA level of 2.0 ng/mL or above, in the context of ADT therapy and in the absence of distant metastasis, be classified as nmCRPC [6]. However, this definition underwent revision in 2015. The updated recommendation posits a lower PSA threshold of 1.0 ng/mL to define nmCRPC [7]. The modifications made by the PCWG3 in their guidelines reflect an ongoing endeavor to optimize the diagnostic criteria for nmCRPC, allowing for enhanced accuracy in identifying cases and potentially improving patient prognosis through timely intervention. Nonetheless, the validity and efficacy of this updated threshold are still under scrutiny and have yet to be unequivocally substantiated.

In this study, we examined the difference in prognosis based on the appearance of metastasis in nmCRPC patients depending on the timing of therapeutic interventions with ARSI. Subsequently, administration of ARSI before PSA levels reached 2.0 ng/mL manifested a notable enhancement in metastasis-free survival relative to cohorts in which administration was deferred. The outcomes of our study advocate for the prompt initiation of ARSI administration, implying that the diagnosis of nmCRPC should ideally precede such interventions. Hence, our findings necessitate a reassessment and corroboration of the PSA threshold of 1.0 ng/mL as a defining criterion for nmCRPC, serving to substantiate the validity of this benchmark in the discernment of nmCRPC. The implications of our results could contribute to refining diagnostic and therapeutic strategies, potentially leading to more timely and effective interventions for patients afflicted with nmCRPC.

Despite the implications of our findings, our study is not without its limitations. First, the retrospective nature of our investigation poses inherent challenges, including potential selection bias. The timing of ARSI administration after PSA increment was mainly at the physician’s discretion, which could make the patient background heterogeneous. Moreover, while we adjusted for lead-time bias in our analyses, other unaccounted biases could influence the outcomes. Our patient cohort, restricted to those who received ARSI, may not be fully representative of the broader nmCRPC population, limiting the generalizability of our results. In this study, we elucidated the upper limit of PSA for ARSI administration (2.0 ng/mL). However, due to the limited number of patients, we could not demonstrate the lower limit of PSA for ARSI administration, but it is essential to avoid overtreatment. Lastly, while our study highlights the significance of early ARSI intervention, future prospective studies with larger and more diverse populations and overall survival are needed to validate our findings.

We conclude that the timely initiation of ARSI is paramount in nmCRPC management. Our findings strongly advocate for consideration of ARSI administration in nmCRPC patients before their PSA levels exceed 2.0 ng/mL. Our results indicated that a PSA threshold of 1.0 ng/mL for nmCRPC definition is more reasonable to administer ARSI without delay, compared to that of 2.0 ng/mL.

## References

[1] Henriquez I, Spratt D, Gómez-Iturriaga A et al (2021) Nonmetastatic castration-resistant prostate cancer: Novel agents to treat a lethal disease. World J Clin Oncol 12(1):6–12 33552935 10.5306/wjco.v12.i1.6 PMC7829629

[2] Smith MR, Cook R, Lee KA et al (2011) Disease and host characteristics as predictors of time to first bone metastasis and death in men with progressive castration-resistant nonmetastatic prostate cancer. Cancer 117(10):2077–2085 21523719 10.1002/cncr.25762 PMC3116053

[3] Hussain M, Fizazi K, Saad F et al (2018) Enzalutamide in Men with Nonmetastatic, Castration-Resistant Prostate Cancer. N Engl J Med 378(26):2465–2474 29949494 10.1056/NEJMoa1800536 PMC8288034

[4] Smith MR, Saad F, Chowdhury S et al (2018) Apalutamide Treatment and Metastasis-free Survival in Prostate Cancer. N Engl J Med 378(15):1408–1418 29420164 10.1056/NEJMoa1715546

[5] Fizazi K, Shore N, Tammela TL et al (2019) Darolutamide in nonmetastatic, castration-resistant prostate cancer. N Engl J Med 380(13):1235–1246 30763142 10.1056/NEJMoa1815671

[6] Scher HI, Halabi S, Tannock I et al (2008) Design and end points of clinical trials for patients with progressive prostate cancer and castrate levels of testosterone: recommendations of the Prostate Cancer Clinical Trials Working Group. J Clin Oncol 26(7):1148–1159 18309951 10.1200/JCO.2007.12.4487 PMC4010133

[7] Scher HI, Morris MJ, Stadler WM et al (2016) Trial Design and Objectives for Castration-Resistant Prostate Cancer: Updated Recommendations from the Prostate Cancer Clinical Trials Working Group 3. J Clin Oncol 34(12):1402–1418 26903579 10.1200/JCO.2015.64.2702 PMC4872347

[8] Maffezzini M, Bossi A, Collette L (2007) Implications of prostate-specific antigen doubling time as indicator of failure after surgery or radiation therapy for prostate cancer. Eur Urol 51(3):605–613 17113217 10.1016/j.eururo.2006.10.062

[9] Pound CR, Partin AW, Eisenberger MA et al (1999) Natural history of progression after PSA elevation following radical prostatectomy. JAMA 281(17):1591–1597 10235151 10.1001/jama.281.17.1591

[10] Cookson MS, Aus G, Burnett AL et al (2007) Variation in the definition of biochemical recurrence in patients treated for localized prostate cancer: the American Urological Association Prostate Guidelines for Localized Prostate Cancer Update Panel report and recommendations for a standard in the reporting of surgical outcomes. J Urol 177(2):540–545 17222629 10.1016/j.juro.2006.10.097

[11] Pfister D, Bolla M, Briganti A et al (2014) Early salvage radiotherapy following radical prostatectomy. Eur Urol 65(6):1034–1043 23972524 10.1016/j.eururo.2013.08.013

[12] Stish BJ, Pisansky TM, Harmsen WS et al (2016) Improved metastasis-free and survival outcomes with early salvage radiotherapy in men with detectable prostate-specific antigen after prostatectomy for prostate cancer. J Clin Oncol 34(32):3864–3871 27480153 10.1200/JCO.2016.68.3425

[13] Saito T, Matsumoto K, Kosaka T et al (2023) Strategy for PSA progression in patients undergoing salvage radiation for biochemical recurrence after radical prostatectomy. Int J Clin Oncol 28(5):707–715 36929093 10.1007/s10147-023-02322-0

[14] Smith MR, Kabbinavar F, Saad F et al (2005) Natural history of rising serum prostate-specific antigen in men with castrate nonmetastatic prostate cancer. J Clin Oncol 23(13):2918–2925 15860850 10.1200/JCO.2005.01.529

